# Assessment of Muscle Function Decline and Cachexia-Related Biomarkers in Hospitalized Oncology Patients: Study Protocol

**DOI:** 10.3390/biomedicines14071504

**Published:** 2026-07-02

**Authors:** Jorge Juan Alvarado-Omenat, Emilio Fonseca-Sánchez, Rocío Llamas-Ramos, Daniel García-García, Marta Correyero-León, Inés Llamas-Ramos

**Affiliations:** 1 Department of Nursing and Physiotherapy, Universidad de Salamanca, Avd. Donantes de Sangre s/n, 37007 Salamanca, Spain; jjao@usal.es (J.J.A.-O.); inesllamas@usal.es (I.L.-R.); 2 Instituto de Investigación Biomédica de Salamanca (IBSAL), P.º de San Vicente, 58-182, 37007 Salamanca, Spain; 3 FisioSport Salamanca, C/Doce de Octunre, nº 2, 37008 Salamanca, Spain; 4 University Hospital of Salamanca, P.º de San Vicente, 182, 37007 Salamanca, Spain; 5 Department of Nursing and Physiotherapy, Universidad de Segovia, Plaza de Colmenares, 40000 Segovia, Spain

**Keywords:** cancer cachexia, sarcopenia, surface electromyography, bioimpedance, hospitalized patients, muscle deterioration

## Abstract

**Background**: Cancer cachexia and sarcopenia are highly prevalent complications affecting up to 50% of patients with cancer and are associated with increased treatment toxicity, poorer functional outcomes, and reduced survival. Early identification of muscle deterioration during hospitalization remains challenging. **Objective**: To evaluate the change in dominant-hand handgrip strength between hospital admission and discharge in hospitalized oncology patients. **Methods**: This prospective longitudinal study will evaluate hospitalized adults with confirmed malignancy and an expected hospital stay of ≥5 days. Daily handgrip strength and sEMG assessments will be performed as exploratory secondary measures to characterize temporal patterns of muscle function during hospitalization. Baseline and discharge evaluations will additionally include bioelectrical impedance analysis, validated patient-reported outcome measures (SARC-F, EORTC QLQ-C30, PSQI), and serum biomarkers related to inflammatory and nutritional status. Linear mixed models will be used to evaluate longitudinal changes and associations between functional, electrophysiological, and biochemical parameters. **Expected results**: The study aims to characterize trajectories of muscle function decline during hospitalization, identify candidate biomarker signatures for cachexia detection, and evaluate neuromuscular fatigue patterns using sEMG. **Conclusions**: This protocol proposes a feasible multimodal framework for monitoring skeletal muscle deterioration during acute oncology hospitalization and may inform future interventional strategies targeting cancer-related cachexia and sarcopenia.

## 1. Introduction

Skeletal muscle deterioration is a critical and common complication in cancer, yet it remains frequently underdiagnosed across different tumor types and disease stages. Cancer cachexia is a multifactorial metabolic syndrome characterized by progressive loss of muscle mass and function, affecting up to 50% of cancer patients and associated with significantly reduced survival [1,2,3,4,5,6].

Its pathophysiology involves a complex interplay between proinflammatory cytokines, an imbalance between protein synthesis and degradation, and systemic metabolic dysfunction. Notably, muscle loss during hospitalization can progress rapidly, with measurable declines occurring within 5 to 10 days [7,8,9].

Current clinical assessment of cancer-related muscle loss largely relies on imaging-based estimates of muscle mass or subjective clinical evaluation. However, these approaches are often impractical for repeated inpatient monitoring, involve radiation exposure, and provide limited insight into neuromuscular function or fatigue mechanisms [2,4,10,11]. Consequently, early detection of functional muscle deterioration during acute hospitalization remains challenging.

The combination of handgrip strength (HGS), surface electromyography (sEMG), and bioimpedance may provide a non-invasive and objective method to assess neuromuscular integrity and detect fatigue patterns, among other parameters; however, some of these techniques remain underutilized in oncology populations, especially during hospitalization. Furthermore, the integration of multiple biomarkers reflecting different pathophysiological axes (inflammatory, nutritional, catabolic) has not been systematically evaluated during short-term inpatient follow-up.

This protocol aims to address these gaps by proposing a comprehensive, multimodal assessment framework specifically designed for hospitalized oncology patients. The approach integrates functional measures (HGS), body composition analysis (bioimpedance), neuromuscular characterization (sEMG), patient-reported outcomes, and serum biomarkers to provide an integrated evaluation of muscle status during hospitalization.

## 2. Objectives

### 2.1. Main Objective

The primary objective is to quantify the change in dominant-hand handgrip strength (HGS) between hospital admission and discharge in hospitalized oncology patients, using the Southampton protocol [12].

### 2.2. Secondary Objectives

Secondary objectives include the characterization of daily trajectories of muscle function, neuromuscular fatigue, body composition, and biomarker profiles during hospitalization.

1. To describe in-hospital trajectories of muscle function through daily assessment of neuromuscular fatigue using sEMG (MDF, MNF, fatigue slope, and, when applicable, time to failure).
2. To determine admission-to-discharge changes in phase angle derived from bioimpedance and in serum indices related to inflammation and nutritional status (Prognostic Nutritional Index, Neutrophil-to-Lymphocyte Ratio, CRP/albumin ratio, CRP/prealbumin ratio).
3. To evaluate associations between muscle deterioration and patient-reported outcomes related to sleep quality, quality of life and symptoms, including the SARC-F, EORTC QLQ-C30, and PSQI scales.

An additional exploratory objective is to identify candidate clinical, functional, electrophysiological, and biochemical variables associated with greater muscle deterioration during hospitalization, with the aim of informing future hypothesis-driven studies.

## 3. Materials and Methods

### 3.1. Study Design

This is a prospective, longitudinal cohort study including hospitalized cancer patients, evaluated from admission to discharge. This protocol was developed in accordance with the STROBE recommendations for observational research. In addition, selected SPIRIT items were considered to enhance transparency and completeness in the reporting of protocol procedures, outcome definitions, and data management plans, adapted to the observational nature of the study. The study will be conducted in accordance with the Declaration of Helsinki and the principles of Good Clinical Practice applicable to non-interventional clinical research. It will take place in the Oncology Ward of the Hospital Clínico Universitario de Salamanca (Salamanca, Spain) during the period March–November 2026.

Trial registration: ClinicalTrials.gov Identifier: NCT07387770, Protocol version: Version 2.0, dated 24 June 2026.

Any protocol modifications affecting study objectives, eligibility criteria, outcome measures, or analytical procedures will be documented, dated, and reported in subsequent publications arising from this project. Study findings will be disseminated through peer-reviewed scientific publications, conference presentations, and academic communications. Results will be reported regardless of the direction or magnitude of the findings.

### 3.2. Participants

#### Sample and Recruitment

The sample will consist of adults (≥18 years) with a confirmed diagnosis of malignant neoplasm admitted to the Oncology Ward of the Hospital Clínico Universitario de Salamanca, recruited consecutively during the period March–November 2026. Eligible patients must have an expected hospital stay of at least 5 days, a functional status of 0–2 according to the Eastern Cooperative Oncology Group (ECOG) scale, and the ability to provide informed consent, complete questionnaires, and perform voluntary contractions required for functional testing. These criteria were established to ensure participant safety, feasibility of assessments, and data quality in the context of repeated bedside neuromuscular evaluations.

Exclusion criteria include contraindications to strength assessment, such as recent fractures, acute musculoskeletal injuries, or pain limiting safe performance of the tests. Patients with severe neurological impairment preventing reliable functional assessment or significant cognitive deficits affecting comprehension or adherence to instructions will also be excluded. In addition, participants with implanted electronic devices will not be included, in accordance with the contraindications or precautions for bioimpedance measurements. Patients with extensive skin lesions or dermatological alterations at electrode placement sites that prevent safe and proper surface electromyography (sEMG) recordings will be excluded.

Cancer stage and current treatment (chemotherapy, immunotherapy, radiotherapy, or supportive care) will be recorded.

A consecutive sampling strategy will be used. Potential participants will be identified in coordination with the medical team, with oncologists referring eligible patients according to the inclusion and exclusion criteria. Nursing staff will also collaborate by providing relevant clinical and care information and by facilitating the scheduling of assessments at times compatible with routine patient care.

A screening log will be maintained throughout the recruitment period to document the number of patients assessed for eligibility, reasons for exclusion, refusal to participate, and recruitment outcomes. When available, basic demographic and clinical information of excluded patients will be summarized in aggregate form to assess the representativeness of the recruited cohort.

The sample size was estimated based on the expected change in HGS, defined as the difference between admission and discharge in the dominant hand, which constitutes the primary outcome. A clinically meaningful difference of 4–5 kg was assumed, based on previous studies in oncology populations, with a standard deviation of 4–5 kg for change in HGS, consistent with prior literature in cancer and comparable adult populations [13].

Using a two-sided significance level of 0.05 and a statistical power of 80%, the estimated minimum sample size ranged between 16 and 25 participants. However, given the exploratory and hypothesis-generating nature of the study, a larger sample size was selected to improve precision and robustness of estimates. A target sample of 50 participants was therefore established. Assuming a potential dropout rate of approximately 10%, the study aims to recruit 55 participants in total. The recruitment period was established based on the expected volume of oncology admissions at the participating institution and previous clinical experience with similar patient populations. Considering the anticipated number of eligible admissions and the target sample size of 55 participants, the planned recruitment window from March to November 2026 is considered feasible. Recruitment feasibility is supported by the high volume of oncology admissions managed by the Medical Oncology Department of the Complejo Asistencial Universitario de Salamanca, a tertiary referral center serving multiple health areas. Although precise screening figures are not available, previous institutional activity suggests that recruitment of 55 participants within the planned recruitment period is achievable.

This approach is consistent with phase II signal-seeking or feasibility study designs, aiming to obtain more stable estimates of HGS changes and variability, reduce the impact of missing data, and enable exploratory multivariable analyses of limited complexity, in line with methodological recommendations for pilot and feasibility studies [14,15,16,17,18]. Daily repeated measurements will not be included in the sample size calculation and will be analyzed as exploratory longitudinal outcomes.

The sample size calculation was based exclusively on the primary outcome and was not intended to support adequately powered predictive multivariable modeling. Accordingly, longitudinal mixed-effects models and multivariable analyses will be considered exploratory and hypothesis-generating, primarily aimed at estimating effect sizes and assessing feasibility. To reduce the risk of overfitting, the number of covariates will be limited a priori to a small set of clinically relevant variables selected based on prior evidence and clinical relevance. Results from these analyses will therefore be interpreted with appropriate caution.

### 3.3. Outcome Measures

#### 3.3.1. Main Outcome Measure

HGS will be measured in both hands at each visit using the standardized Southampton protocol [12], performing three attempts per hand and recording the maximum value for each hand [19]. This bilateral measurement reduces the risk of underestimating true maximal strength, as a substantial proportion of individuals may achieve their highest value in the non-dominant hand [20]. To avoid multiplicity of endpoints and ensure consistency with regulatory recommendations, including those of the U.S. Food and Drug Administration (FDA) [21], the primary outcome is prespecified as the absolute change in dominant-hand HGS between hospital admission and discharge, calculated as the difference between the maximum dominant-hand value recorded at discharge and the corresponding baseline measurement. Daily HGS measurements obtained throughout hospitalization will be analyzed as secondary exploratory outcomes to characterize temporal patterns of muscle deterioration.

#### 3.3.2. Secondary Outcome Measure

The non-dominant hand and bilateral analyses, as well as sEMG parameters, bioimpedance, biomarkers, and questionnaires, will be considered secondary/exploratory outcomes, consistent with the use of exploratory endpoints for hypothesis generation without confirmatory intent [17,22].

#### 3.3.3. Pre-Specified Covariates (For Adjustment)

To minimize overfitting in multivariable models, a limited set of clinically relevant predictors will be pre-specified before the start of the analysis. These were selected based on their widespread use as prognostic or confounding factors in oncology: age, sex, functional status measured by ECOG, cancer histological type, presence of major comorbidity, and length of hospital stay [23,24,25]. Age and ECOG functional status have been consistently associated with survival and treatment tolerance in multiple oncology cohorts, with significantly unfavorable hazard ratios for older patients and ECOG 2–4 compared to ECOG 0–1 [24,25,26]. Sex and cancer histological type are routinely used as adjustment variables to capture biological differences and treatment response across subgroups [23,24,25]. Finally, the burden of major comorbidity has been linked to both higher dropout rates in clinical trials and poorer overall survival, justifying its inclusion as an adjustment covariate in prognostic and functional response models [27,28]. Length of hospital stay was additionally included because prolonged hospitalization may increase exposure to physical inactivity, systemic inflammation, nutritional deterioration, and other factors associated with accelerated muscle loss.

### 3.4. Instruments

#### Questionnaires

SARC-F

The SARC-F [29] is a brief screening questionnaire consisting of five items assessing strength, assistance in walking, rising from a chair, climbing stairs, and falls. Each item is scored from 0 to 2, giving a total score range of 0 to 10. A score of ≥4 is considered indicative of sarcopenia risk and suggests the need for a more detailed musculoskeletal assessment.

EORTC QLQ-C30

The EORTC QLQ-C30 [30] is a standardized 30-item questionnaire developed to assess health-related quality of life in oncology patients. It includes functional scales (physical, emotional, cognitive, social, and role), symptom scales, and a global health/quality of life measure. Specifically, it comprises 5 functional scales (physical, role, emotional, cognitive, and social), 3 symptom scales (fatigue, pain, nausea/vomiting), 1 global health/quality of life scale, and additional individual symptom items.

Scores are linearly transformed to a 0–100 scale. For functional and global scales, higher scores indicate better functioning or quality of life. For symptom scales, higher scores indicate greater symptom severity (worse clinical status). A change of ≥10 points is generally considered clinically meaningful in many oncology contexts.

Pittsburgh Sleep Quality Index (PSQI)

The Pittsburgh Sleep Quality Index (PSQI) [31] assesses subjective sleep quality over the past month through 19 items grouped into 7 components (subjective quality, latency, duration, habitual efficiency, disturbances, use of medication, and daytime dysfunction).

Each component is scored from 0 to 3, yielding a global score ranging from 0 to 21. Higher scores indicate poorer sleep quality. A cutoff point >5 is commonly used to identify clinically relevant poor sleep quality.

It is acknowledged that the PSQI evaluates sleep quality over the previous month; repeating the assessment at discharge provides a mixed estimate of the prior period and the partial impact of hospitalization. To contextualize changes, qualitative comments on sleep specifically during the hospital stay will be recorded.

The PSQI is intended to provide a standardized assessment of baseline sleep quality and sleep-related vulnerability rather than a direct measure of short-term hospitalization-related sleep changes. Consequently, PSQI findings will be interpreted within the context of its established one-month recall period.

Handgrip Strength (HGS)

Grip strength will be assessed using the Camry EH101 hand dynamometer (Zhongshan Camry Electronic Co. Ltd., Zhongshan, China), a low-cost, portable device that has demonstrated excellent intra- and inter-instrument reliability (ICC > 0.94) and concurrent validity against the Jamar^®^ dynamometer (gold standard, Warrenville, IL, USA), with minimal systematic bias (0.5–1.4 kg) and narrow limits of agreement in adult and geriatric clinical populations, including cancer patients and healthy individuals [32].

HGS will be measured following the standardized bilateral positioning and measurement protocol from the University of Southampton [12,33]. During the assessment, participants will be seated with their back supported, shoulder in a neutral position, elbow flexed at 90°, and wrist extended between 0° and 30°. The procedure consists of performing three maximal voluntary contractions per limb, with 60 s recovery periods between efforts to prevent cumulative fatigue. For the final analysis, the highest value achieved across the three repetitions for each hand will be recorded.

Bioelectrical Impedance Analysis (BIA)

Body composition will be assessed using bioelectrical impedance analysis (BIA) with the Tanita MC-580 device (Tanita Corp., Tokyo, Japan), which employs multifrequency BIA technology like other clinically validated models from the same manufacturer and has been previously validated against Dual-Energy X-ray Absorptiometry (DXA) (Marlborough, MA, USA). These devices show high correlations (r ≈ 0.85) and technical errors of approximately 1.5–2.5% for total and segmental body fat in healthy adults and individuals with obesity [34,35]. Although this method provides acceptable accuracy for clinical and research purposes, it remains an indirect approach compared with four-compartment models, and therefore its limitations must be acknowledged, particularly in clinical populations.

Measurements will be performed following a standardized protocol. Participants will be instructed to void approximately 30 min before assessment and to rest in supine position for 15 min prior to measurement to minimize variability related to fluid redistribution. Primary electrical parameters, including resistance (R) and reactance (Xc), will be recorded, and derived indices such as phase angle, skeletal muscle mass, fat-free mass, fat mass, and total body water will be calculated. Phase angle will be considered the primary impedance-derived parameter due to its relative stability and lower sensitivity to acute fluid shifts compared to other indices derived.

To improve interpretability, hydration-related and clinical factors will be systematically recorded whenever available, including peripheral edema, ascites, recent intravenous fluid administration, fasting status, and other relevant conditions that may affect fluid distribution. Patient posture during measurement will also be documented, and all assessments will be conducted in a standardized position whenever clinically feasible.

Given the potential influence of acute fluid shifts in hospitalized oncology patients, BIA-derived body composition estimates will be interpreted as exploratory indicators rather than definitive measures of skeletal muscle mass. In this context, particular attention will be given to phase angle, as it may provide a more stable indicator of cellular integrity and nutritional status than other impedance-derived variables. However, all phase angle interpretations will be considered alongside the patient’s clinical condition and hydration status.

Body Weight, Body Mass Index, and Dietary Intake

Body weight (kg) and height (m) will be recorded at hospital admission. Body Mass Index (BMI) will be calculated as weight (kg) divided by height squared (m^2^). Body weight will be reassessed at discharge and monitored during hospitalization whenever routinely available through clinical records.

Dietary intake during hospitalization will be documented using standardized nursing and clinical nutrition records when available. Intake will be estimated as the percentage of meals consumed and categorized as adequate (>75%), moderate (50–75%), or insufficient (<50%) intake. These variables will be considered exploratory indicators of nutritional status and potential contributors to muscle deterioration.

Additional Clinical Variables

To provide a broader characterization of factors potentially influencing muscle deterioration, the following variables will be recorded whenever available from the medical record: cancer stage, reason for hospital admission, recent anticancer treatment and treatment timing, corticosteroid exposure, opioid or sedative medication use, nutritional support interventions, clinically significant infection or inflammatory complications, anemia status, mobility restrictions, and hydration-related factors, including edema, ascites, and intravenous fluid administration.

Surface Electromyography (sEMG)

Electromyographic activity was recorded using the wireless Shimmer3 system, integrated into the mDurance platform [36]. Signals were acquired at a sampling frequency of 1000 Hz, ensuring adequate temporal resolution for amplitude and frequency parameter analysis.

A band-pass filter between 10 and 500 Hz was applied to remove low-frequency components and high-frequency noise, along with a 50 Hz notch filter to suppress electrical interference. Acquisition and preprocessing parameters were kept constant across all assessments to ensure intra- and inter-subject comparability.

Skin preparation and electrode placement were performed according to SENIAM guidelines. Electrodes were positioned on the vastus lateralis, vastus medialis, and upper trapezius. The vastus lateralis and medialis were selected due to their key role in functional mobility and load transfer during activities of daily living, as well as their documented sensitivity to accelerated muscle loss in prolonged disuse and bed rest contexts [37,38,39]. The upper trapezius was chosen as an accessible postural muscle relevant during prolonged sitting, allowing evaluation of fatigue patterns distinct from those of the lower limbs [40,41].

Lower limb protocol: Three maximal voluntary contraction (MVC) attempts of 3 s each will be performed, with 2 min rest intervals between attempts. Subsequently, an isometric contraction at 65% of MVC will be maintained using biofeedback via a portable dynamometer until muscle failure, defined as the inability to maintain the target intensity (±5–10%) for more than 3–5 consecutive seconds despite visual/auditory feedback [42,43].

Upper trapezius protocol: Three MVC attempts of 3 s each will be performed, with 2 min rest intervals between attempts. This will be followed by an isometric contraction at 65% of MVC using biofeedback via a portable dynamometer until muscle failure.

Participants will perform a sustained isometric contraction at 65% of their maximal voluntary contraction (MVC). Reduction to 50% MVC will only be considered when participants are unable to safely maintain the target force level during familiarization, report excessive fatigue, pain, or discomfort, or when the research team determines that continuation at 65% MVC may compromise participant safety [43,44,45,46,47,48]. The contraction intensity selected and the reason for any modification will be documented for each assessment. To ensure isometric contractions at 65% MVC in the vastus lateralis and medialis (quadriceps) and upper trapezius during the muscle assessment protocol, the portable ActivForce 2 dynamometer (ActivForce, LLC, San Diego, CA, USA) will be used. This device provides real-time feedback via a mobile app with precise force measurements (intra- and inter-rater ICC > 0.85–0.99; concurrent validity r = 0.89–0.93 vs. microFET2 in isometric shoulder and knee movements), allowing patients to adjust intensity objectively and safely [49,50].

Participants may stop the contraction at any time if they experience pain, dyspnea, dizziness, or subjective discomfort; these events will be systematically recorded. In patients with clinical limitations, the protocol may be adjusted to 50% MVC to ensure safety without compromising the acquisition of fatigue parameters.

Surface electromyographic signals were analyzed following methodologies previously described in the literature [51]. In the time domain, the root mean square (RMS) was calculated and normalized to the value obtained during maximal voluntary contraction (%MVC), to allow intra- and inter-subject comparisons.

In the frequency domain, median frequency (MDF) and mean frequency (MNF) were estimated using the fast Fourier transform (FFT), applying consistent analysis windows throughout the signal to ensure spectral stability.

Neuromuscular fatigue indices were calculated as follows: Temporal slope of MDF during sustained contraction; Final/initial RMS ratio, as an indicator of changes in muscle activation; Time to failure, defined as the moment when the participant was no longer able to maintain the target force level; failure was considered when force decreased persistently by more than 5–10% below 65% of MVC during the isometric contraction [43,44].

During data acquisition, real-time visual inspection was performed to detect artifacts (movement, electrical interference, or loss of electrode contact), complemented by a systematic post-acquisition review to ensure the quality and consistency of the recordings prior to final analysis.

sEMG Acquisition and Signal Processing Procedures

All assessments will be performed with participants seated comfortably in a chair or hospital bed with back support. For quadriceps recordings, hips and knees will be positioned at approximately 90° flexion whenever clinically feasible, while for upper trapezius recordings, participants will remain seated with arms relaxed alongside the body. Standardized positioning will be maintained across sessions whenever possible.

Surface electromyography (sEMG) electrode placement will follow SENIAM recommendations. Bipolar Ag/AgCl surface electrodes will be positioned parallel to muscle fiber orientation with an inter-electrode distance of 20 mm. Skin preparation will include shaving, when necessary, gentle abrasion, and cleaning with alcohol to reduce impedance.

Force measurements and sEMG signals will be recorded simultaneously and synchronized through the acquisition software. Raw signals will be visually inspected prior to analysis to identify artifacts and signal abnormalities.

Signal processing will include band-pass filtering, full-wave rectification, and frequency-domain analysis using Fast Fourier Transform (FFT) techniques. Spectral analyses will be performed using Hamming windows of 1024 samples with 50% overlap. Mean and median frequency parameters will be calculated from consecutive one-second epochs during the sustained contraction task.

Prior to analysis, all recordings will undergo visual inspection and quality-control procedures. Signal segments will be excluded if they exhibit evidence of electrode displacement, signal saturation, excessive baseline noise, movement artifacts, abrupt signal discontinuities, or other technical abnormalities that may compromise signal interpretation. When multiple epochs are available, only artifact-free segments meeting predefined quality criteria will be included in the final analysis.

To ensure reliability in the bedside hospital setting, all evaluators will undergo standardized training, and all procedures will follow predefined operating protocols. Electrode placement sites will be systematically documented to ensure consistent positioning across repeated measurements.

Safety Monitoring and Adverse Event Recording

All assessments will be performed only when participants are considered clinically stable by the attending medical team. Prior to each testing session, patients will be screened for contraindications, including acute clinical deterioration, uncontrolled pain, severe fatigue, hemodynamic instability, active infection requiring urgent intervention, or any condition that may compromise safe participation.

Adverse events occurring during or immediately after testing will be documented, including dizziness, excessive fatigue, pain exacerbation, musculoskeletal discomfort, cardiovascular symptoms, falls, or any unexpected clinical event. The severity, duration, and relationship to the assessment procedure will be recorded.

Testing will be immediately discontinued if participants report intolerable discomfort, request termination, exhibit signs of excessive fatigue, develop cardiovascular symptoms, or if the research team determines that continuation may compromise safety. Participant safety will always take precedence over protocol completion.

Serum biomarkers—Basic Panel

Laboratory assessments will be performed at hospital admission and discharge. In cases where daily blood tests are required for clinical reasons, these additional results will be incorporated into the longitudinal analysis.

The following parameters will be collected: albumin, prealbumin, C-reactive protein (CRP), creatinine, complete blood count, lipid profile, and glucose. From these values, several derived indices will be calculated, including the Prognostic Nutritional Index (PNI) [52,53,54,55,56], the neutrophil-to-lymphocyte ratio (NLR) [57,58,59,60], the CRP/albumin ratio [61,62], and the CRP/prealbumin ratio [63], as integrated markers of inflammatory, nutritional, and catabolic status.

Additionally, Growth Differentiation Factor 15 (GDF-15) will be determined as an exploratory biomarker of cancer-related cachexia. GDF-15 has been associated with anorexia, systemic catabolism, involuntary weight loss, and adverse clinical outcomes in oncology populations, making it a promising candidate marker for identifying patients at risk of accelerated muscle deterioration.

### 3.5. Procedure

Following the identification of eligible patients by the Oncology team and verification of inclusion and exclusion criteria, participants will receive detailed information about the study and will provide written informed consent prior to any study-specific procedures.

Assessments will be conducted in the patient’s room, either self-administered or assisted by a researcher following a standardized script to ensure consistency in data collection (Table 1).

On the day of admission (Day 1), a comprehensive baseline assessment will be performed, including questionnaires (SARC-F, EORTC QLQ-C30, and PSQI), HGS measurement, body composition assessment using bioelectrical impedance, electromyographic recording of selected muscles, and blood sampling for serum biomarker determination.

From Day 2 until the penultimate day of hospitalization, daily functional assessments will be performed, consisting of HGS measurement and an sEMG protocol for neuromuscular fatigue characterization. These assessments will be scheduled in coordination with the clinical team to avoid interference with medical or therapeutic procedures.

On the day of hospital discharge, the full assessment performed at admission will be repeated, allowing analysis of absolute admission-to-discharge changes in both primary and secondary outcomes.

All evaluations will follow previously described standardized procedures, including quality control measures, periodic equipment calibration, and systematic recording of clinical events that may influence measurements. The assessment schedule (Table 1) is designed to integrate into routine clinical practice, minimizing patient burden while always ensuring safety.

### 3.6. Criteria for Suspending/Omitting Measurements

Measurements will be suspended in the presence of fever > 38 °C, symptomatic hypotension, moderate-to-severe pain, nausea, or clinical events that compromise participation. The possibility of omitting measurements on specific days is considered part of the ethical and pragmatic management of research in hospitalized oncology patients.

### 3.7. Data Collection and Quality Assurance (STROBE: “Bias” and “Data Quality”)

Data will be collected using standardized forms and subsequently entered into a REDCap electronic database, ensuring traceability and version control. A double-entry procedure will be applied to minimize transcription errors. Measurement equipment, including the dynamometer and electromyography system, will be calibrated monthly according to internal technical control protocols. Evaluators will receive standardized training prior to study initiation, and inter-rater reliability will be verified using intraclass correlation coefficients (ICC) greater than 0.90 to reduce measurement bias and ensure the methodological quality of the study.

### 3.8. Ethical Considerations

The protocol was approved by the Research Ethics Committee (23/06/2025 CEIm Ref. 2025/07). Due to the vulnerable nature of this population, the consent process will emphasize clear and understandable language, adequate time for decision-making, voluntariness without impact on clinical care, the right to withdraw participation at any time, and the presence of an impartial witness in cases of limited literacy. Data will be anonymized and stored in encrypted databases in accordance with Regulation (EU) 2016/679 (GDPR), Organic Law 3/2018 on the Protection of Personal Data and guarantee of digital rights, and Law 14/2007 on Biomedical Research.

### 3.9. Statistical Analysis

The primary endpoint is the absolute change in dominant-hand handgrip strength (HGS) between hospital admission and discharge. The primary analysis will consist of a within-subject comparison of admission and discharge HGS values. Mean change scores and corresponding 95% confidence intervals will be reported. Statistical significance will be assessed using a paired *t*-test for normally distributed data or the Wilcoxon signed-rank test when normality assumptions are not met. All tests will be two-sided, with a significance level of *p* < 0.05. Exploratory longitudinal analyses will be performed using linear mixed-effects models to characterize daily trajectories of handgrip strength (HGS) and sEMG parameters during hospitalization while accounting for repeated measurements within participants and unequal numbers of observations across individuals. These analyses are intended to describe temporal patterns of muscle function and neuromuscular fatigue and are considered secondary and exploratory. They do not constitute the primary analysis of the study. Missing data due to discharge, clinical deterioration, medical procedures, symptom burden, or death will be handled under the missing-at-random (MAR) assumption inherent to mixed-effects modeling. The frequency and reasons for missing observations will be systematically documented across all outcome domains.

The primary endpoint is the absolute change in dominant-hand handgrip strength (HGS) between hospital admission and discharge. The primary analysis will consist of a within-subject comparison of admission and discharge HGS values. Mean change scores and corresponding 95% confidence intervals will be reported. Statistical significance will be assessed using a paired *t*-test for normally distributed data or the Wilcoxon signed-rank test when normality assumptions are not met. All tests will be two-sided, with a significance level of *p* < 0.05.

Associations between functional outcomes, sEMG parameters, and biomarkers will be explored using multivariable regression models adjusted for pre-specified covariates (age, sex, ECOG performance status, cancer type, comorbidity burden, and length of hospital stay). Given the exploratory nature of the study and the planned sample size, these analyses will be interpreted cautiously and will primarily serve to estimate effect sizes and identify candidate variables for future confirmatory studies. The contraction intensity used during each sEMG fatigue assessment (65% or 50% MVC) will be recorded and considered during data interpretation. Because contraction intensity may influence neuromuscular fatigue parameters, exploratory analyses will account for intensity level and, when sample size permits, stratified analyses will be performed. No direct comparison between intensity conditions is planned, and results obtained under different intensity levels will be interpreted cautiously.

Participants with both admission and discharge handgrip strength (HGS) measurements will be included in the primary endpoint analysis. The frequency and reasons for missing discharge assessments, including early discharge, transfer to another facility, clinical deterioration, or death, will be systematically documented.

Sensitivity analyses will compare baseline demographic and clinical characteristics between participants with complete and incomplete primary endpoint data. When appropriate, exploratory analyses based on the last available HGS measurement obtained during hospitalization will be conducted to assess the robustness of the primary findings. Results from these analyses will be interpreted cautiously due to the potential for informative missingness.

Hydration-related variables, including edema, ascites, and recent intravenous fluid administration, will be considered in the interpretation of bioelectrical impedance analysis (BIA)-derived outcomes and may be incorporated into sensitivity analyses when clinically relevant. Additional clinical variables may also be included in exploratory or sensitivity analyses when justified and supported by sufficient data availability.

Sensitivity analyses will be performed when substantial missing data or potential sources of bias are identified, to assess the robustness of the findings. Attention will be given to variables in which missingness may be related to clinical status or disease severity. Biomarker measurements obtained only when clinically indicated will be analyzed descriptively and interpreted with caution due to potential non-random availability across participants.

Overall, all association analyses are exploratory in nature and intended to generate hypotheses and provide preliminary estimates of potential relationships for future confirmatory studies. The number of repeated assessments may vary among participants depending on hospitalization duration and clinical feasibility.

### 3.10. Rationale for Selection of Outcome Measures and Biomarkers

The selection of outcome measures and biomarkers in this study was guided by their clinical validity, sensitivity to detect early changes in muscle function and nutritional status, feasibility in acutely hospitalized oncology patients, and prior use in cachexia and sarcopenia research.

Handgrip strength (HGS) was chosen as the primary functional outcome due to its strong association with global muscle strength, functional status, treatment tolerance, and clinical outcomes in oncology populations. It is a simple, reliable, and validated bedside measure that is sensitive to short-term changes during hospitalization.

Surface electromyography (sEMG) was included to assess neuromuscular activation patterns and fatigue development, providing information on muscle function beyond maximal force production. This allows evaluation of muscle quality and neuromuscular efficiency, which are particularly relevant in early stages of muscle deterioration.

Bioelectrical impedance analysis (BIA) was selected as a non-invasive and feasible method for assessing body composition and detecting changes in fat-free mass and phase angle, both of which are associated with nutritional status and prognosis in cancer patients.

Inflammatory and nutritional biomarkers, including C-reactive protein (CRP), albumin, prognostic nutritional index (PNI), and neutrophil-to-lymphocyte ratio (NLR), were included due to their established relevance in oncology-related cachexia, systemic inflammation, and muscle wasting processes.

Validated patient-reported outcome measures (SARC-F, EORTC QLQ-C30, and PSQI) were incorporated to capture sarcopenia risk, functional status, quality of life, and sleep quality, thereby providing a comprehensive patient-centered assessment.

Overall, the combined use of these tools enables an integrated evaluation of muscle function, neuromuscular fatigue, biochemical status, and patient-reported outcomes, which is essential for understanding early muscle deterioration in hospitalized oncology patients.

### 3.11. Rationale for Muscle Selection

Although handgrip strength is the primary functional outcome, surface electromyography was performed in the vastus lateralis, vastus medialis, and upper trapezius because these muscles represent key functional regions commonly affected by hospitalization-related inactivity. The quadriceps muscles play a central role in mobility, sit-to-stand performance, and functional independence and are particularly susceptible to rapid disuse atrophy. The upper trapezius was included as a representative postural muscle frequently involved in daily activities and prolonged bed rest adaptations. Together, these muscle groups provide complementary information regarding generalized neuromuscular deterioration that may not be fully captured by handgrip strength alone.

## 4. Study Relevance

The protocol will provide characterization of trajectories of muscle deterioration; exploratory evaluation of biomarker profiles associated with muscle deterioration; neuromuscular fatigue parameters to differentiate muscle loss from dysfunction; relationships between sleep, symptom burden, and function; and evidence regarding the feasibility of multimodal assessment in real-world hospitalization settings. The findings of this study may help identify candidate variables associated with hospitalization-related muscle deterioration and provide preliminary data to support the design of future prospective and hypothesis-driven studies investigating potential determinants and mechanisms of muscle deterioration in hospitalized oncology patients.

Repeated fatigue assessments may impose an additional burden on some hospitalized oncology patients. Potential sources of measurement variability include learning effects, day-to-day fluctuations in clinical status, and incomplete protocol adherence due to fatigue or medical complications. For this reason, feasibility outcomes, protocol adherence, adverse events, and reasons for missed or interrupted assessments will be systematically recorded to inform future optimization of the assessment strategy.

The findings of this study may help identify candidate variables associated with hospitalization-related muscle deterioration and provide preliminary data to support the design of future prospective studies aimed at developing and validating predictive models.

## 5. Clinical Implications and Future Research Directions

Early identification of muscle deterioration may facilitate individualized exercise prescription, precision nutrition, targeted pharmacological development, and optimization of outcomes in clinical trials. Future directions include post-discharge follow-up, integration of imaging (CT/DXA), molecular biomarkers (microRNAs, GDF-15), and specific interventional trials.

The feasibility of repeated sEMG assessments in hospitalized oncology patients represents an important aspect of the present protocol. Surface electromyography was selected because it is non-invasive, portable, relatively rapid to perform, and suitable for bedside evaluation. Nevertheless, factors such as clinical instability, severe fatigue, acute complications, diagnostic procedures, or patient refusal may occasionally limit daily assessments. Therefore, adherence to the planned assessment schedule and reasons for missed measurements will be systematically recorded to evaluate the practical implementation of the protocol in routine clinical settings.

Future studies may benefit from incorporating brief hospital-specific daily assessments of symptoms, sleep quality, fatigue, mobility, and nutritional intake to better characterize short-term fluctuations during hospitalization while maintaining acceptable participant burden.

## 6. Limitations and Considerations

The heterogeneity of cancer types, disease stages, and ongoing treatments may introduce variability in muscle function trajectories. In addition, hospital-related factors such as acute illness severity, medication effects, and differences in mobility restrictions may influence functional measurements.

Eligibility criteria, including an Eastern Cooperative Oncology Group (ECOG) performance status of 0–2, an expected hospital stay of at least 5 days, and the ability to perform voluntary neuromuscular assessments, were defined to ensure feasibility and patient safety but may lead to underrepresentation of the most frail patients, including those with severe functional impairment, advanced disease burden, or marked clinical instability. This may limit the generalizability of the findings to the broader population of hospitalized oncology patients.

To mitigate sources of variability, several strategies will be implemented, including strict eligibility criteria, statistical adjustment for relevant covariates, exclusion of extreme cases when appropriate, systematic clinical documentation of hydration status, and quality assurance procedures such as device calibration, standardized training, and inter-rater reliability monitoring. Despite these measures, the planned sample size may not allow simultaneous adjustment for all potentially relevant determinants of muscle deterioration, and thus multivariable analyses should be interpreted as exploratory and hypothesis-generating. Similarly, the sample size is insufficient for the development or validation of predictive or risk stratification models.

Missing data are expected due to clinical instability, symptom burden, hospital discharge, competing medical priorities, or death. Although linear mixed-effects models can accommodate incomplete repeated-measures data under a missing-at-random assumption, the possibility of informative missingness cannot be excluded and may influence the interpretation of longitudinal analyses. In addition, daily assessments may not be feasible for all participants throughout hospitalization, contributing to irregular measurement intervals and incomplete trajectories.

Some patient-reported outcome measures, particularly the Pittsburgh Sleep Quality Index, were originally designed to assess health status over longer recall periods and may therefore have limited sensitivity to short-term changes occurring during hospitalization. While these instruments provide valuable contextual information, they may not fully capture day-to-day fluctuations in symptoms or functional status in acute inpatient settings.

Bioelectrical impedance analysis (Tanita MC-580) provides a practical bedside method for assessing body composition but remains a doubly indirect technique that may over- or underestimate body composition (±2–3%) compared with four-compartment models, particularly in patients with fluid imbalance or severe cachexia. Although it shows acceptable correlation with dual-energy X-ray absorptiometry (r ≈ 0.85) and is suitable for longitudinal monitoring, its accuracy may be affected by hydration disturbances, edema, ascites, and intravenous fluid administration, which are common in hospitalized oncology patients. Therefore, BIA-derived estimates of muscle mass should be interpreted as exploratory indicators rather than definitive measures of body composition.

Because discharge assessments may be unavailable in patients experiencing clinical deterioration, transfer, or death, informative missingness cannot be completely excluded and may influence the interpretation of the primary outcome.

## 7. Conclusions

This protocol provides a feasible and methodologically rigorous framework for the multimodal assessment of skeletal muscle deterioration in hospitalized cancer patients. Its implementation adheres to ethical standards and prioritizes real-world clinical applicability. It is expected to improve understanding of the dynamics of muscle deterioration during the acute phase and generate evidence to inform future trials targeting cancer-related cachexia and sarcopenia during hospitalization.

## Figures and Tables

**Table 1:** Assessment schedule.

Assessment	Screening	Admission	Daily	Discharge
Eligibility criteria	X			
Informed consent		X		
Demographic and clinical data		X		
Cancer characteristics		X		
Weight		X	X	X
BMI		X		X
Dietary intake			X	
Handgrip strength		X	X	X
sEMG assessment		X	X	X
Bioelectrical impedance analysis		X	X	X
Fatigue questionnaires		X	X	X
Quality of life measures		X		X
Sleep assessment (PSQI)		X		X
Biomarkers/laboratory parameters		X	X	X
Adverse events			X	X
Hydration-related variables		X	X	X

## Data Availability

No study dataset has been generated at the time of publication of this protocol. Following study completion, de-identified data supporting the findings may be made available by the corresponding author upon reasonable request.

## Abbreviations

The following abbreviations are used in this manuscript:

BMI	Body Mass Index
CEIm	Comité de Ética de la Investigación con medicamentos
CRP	C-reactive protein
DXA	Dual-energy X-ray absorptiometry
ECOG	Eastern Cooperative Oncology Group
EORTC QLQ-C30	European Organisation for Research and Treatment of Cancer Quality of Life Questionnaire
FFT	Fast Fourier Transform
GDPR	General Data Protection Regulation
HGS	Handgrip strength
ICC	Intraclass Correlation Coefficient
MDF	Median Frequency
MNF	Mean Frequency
MVC	Maximal Voluntary Contraction
NLR	Neutrophil-to-Lymphocyte Ratio
PNI	Prognostic Nutritional Index
PSQI	Pittsburgh Sleep Quality Index
REDCap	Research Electronic Data Capture
RMS	Root Mean Square
SARC-F	Strength, Assistance in walking, Rise from a chair, Climb stairs, Falls
sEMG	Surface Electromyography
SPIRIT	Standard Protocol Items: Recommendations for Interventional Trials
STROBE	Strengthening the Reporting of Observational Studies in Epidemiology
